# Genetic distribution of selected obesity-related SNVs in the *FTO*, *DRD2*, *MC4R*, *FABP2*, *ADRB2*, and *SH2B1* genes in a Mexico city population

**DOI:** 10.3389/fgene.2026.1784283

**Published:** 2026-04-10

**Authors:** Carlos Perezcano, Andrea Sánchez, Gerardo Cendejas, Francisco Borrayo, Mariana Pérez-Coria, Gerardo Pérez-Hernández

**Affiliations:** 1 Center for Research in Precision Medicine and Clinical Genomics – Genomics 360, Mexico City, Mexico; 2 Departamento de Ciencias Naturales, Universidad Autónoma Metropolitana, Unidad Cuajimalpa, Ciudad de México, Mexico

**Keywords:** FTO gene, genetic variation in Mexican population, *MC4R* gene, obesity-related genes, obesity-related variants, population genetics, single nucleotide variants (SNVs)

## Abstract

**Introduction:**

Obesity represents a significant health challenge worldwide, with an increasing trend and high prevalence in Mexico. This metabolic disease, characterized by an increase in body mass index (BMI), leads to abnormal fat accumulation that contributes to several pathologies, and is determined by a complex interaction between diet, lifestyle and genetic factors, such as single nucleotide variants (SNVs) that may influence appetite regulation, adipose tissue function, energy metabolism, reward mechanisms, motivation, food intake behavior control, energy expenditure, fatty acid transport, lipid accumulation, lipolysis, insulin sensitivity, glucose metabolism, among other biochemical processes. However, the frequency of obesity-associated genetic variants remains poorly characterized in Mexican populations–which are highly admixed–as demonstrated by population genetic studies which have established the influence of this admixture in the prevalence and distribution of obesity-related SNVs.

**Methods:**

This descriptive population genetic study aimed to characterize the genotype and allele frequency distributions of six SNVs previously associated with obesity and metabolic traits: *FTO* rs9939609, *ANKK1/DRD2* rs1800497, *MC4R* rs17782313, *FABP2* rs1799883, *ADRB2* rs1042714, and *SH2B1* rs4788102 through a cross-sectional design conducted in a cohort from Mexico City (*N* = 129).

**Results and Discussion:**

The genotyping results revealed substantial variability in allelic frequencies across the analyzed variants, with *SH2B1* rs4788102 showing the highest minor allele frequency (MAF) (0.41), followed by *ANKK1/DRD2* rs1800497 (0.32) and *FTO* rs9939609 (0.31), whereas *MC4R* rs17782313 presented the lowest MAF (0.12). All SNVs were in HWE (*p* > 0.05). Understanding the prevalence of these obesity-related genetic markers in Mexican populations provides a reference for future genomic and nutrigenomic studies, and may support the development of personalized prevention strategies contributing to deeper insight into the molecular basis of metabolic diseases in Mexico, given the serious public health challenge they represent.

## 1 Introduction

Obesity represents a significant public health challenge, and is one of the leading causes of morbidity and mortality in the world. It is defined as a body mass index (BMI) ≥ 30 kg/m^2^ and is characterized by excess adiposity associated with increased risk of metabolic and cardiovascular complications (Mahadevan and Ali, 2016). In Mexico, the situation is particularly critical, the country ranks second among the Organization for Economic Cooperation and Development (OECD) in obesity prevalence, only behind the United States (Campos-Vazquez RM, 2019). Additionally, the country has an increasing trend in overweight and obesity, with one of the worldwide highest prevalence rate at 75% (INSP) (Shamah, 2016). This growing epidemic in Mexico is driven by a multifactorial interplay of behavioral, environmental, physiological, sociocultural, and economic factors, as well as a strong genetic component, with heritability estimates reaching 70% (Pineda et al., 2023). In addition, Mexico has undergone a rapid epidemiological transition, shifting from traditional diets to ultra-processed foods high in sugar and fats. This dietary transformation, coupled with socioeconomic factors that limit access to healthy foods and opportunities for physical activity, exacerbates the obesity crisis (Campos-Vazquez RM, 2019). The high prevalence of obesity in Mexico underscores the need to explore the interplay between genetic and epigenetic factors. Among the various factors associated with obesity, genetics has shown that single nucleotide variants (SNVs) in various genes can contribute to the development of multiple obesity-related diseases. In recent decades, genome-wide association studies (GWAS) have identified multiple candidate genes and SNVs linked to obesity in European and Asian populations (Visscher et al., 2017). Nevertheless, there remains a significant gap in understanding how these findings apply to Mexican populations, particularly given the genetic heterogeneity of the Mexican population. Therefore, it is crucial to expand population genetic studies in Mexican cohorts, not only to validate whether known obesity-associated variants are equally relevant, but also to uncover population-specific genetic markers associated with obesity-related traits. Such efforts are essential for developing nutrigenomic precision medicine approaches, improving risk prediction, and implementing targeted public health strategies in one of the world’s most affected populations.

## 2 Role of genetic factors and common SNVs related to obesity

### 2.1 Fat mass and obesity-associated (*FTO*)

The *FTO* gene was the first obesity susceptibility-related gene identified through genome-wide association studies (GWAS) (Frayling et al., 2007). The protein encoded by this gene has been identified as an RNA demethylase associated with the demethylation of N6-methyladenosine, the most common RNA methylation. Different SNVs present in this gene have been strongly associated with increased BMI, increased obesity risk and type 2 diabetes across diverse populations, including European, Asian, and Mexican-Mestizo groups (Meyre et al., 2009; Yajnik et al., 2009; Astiazaran-Garcia et al., 2017). Among all of these, rs9939609 stands out as one of the most widely studied (Huang et al., 2023). Individuals who are homozygous for the A risk allele of rs9939609 tend to have increased energy and fat intake, increased appetite, and reduced satiety (Tanofsky-Kraff et al., 2009). Other important SNVs, such as rs17817449, rs12149832, and rs1421085, also contribute to obesity susceptibility and often occur in haplotypes that regulate gene expression via intronic regions (Meyre et al., 2009; Yajnik et al., 2009; Vasan et al., 2012; Astiazaran-Garcia et al., 2017).

Another important variant in the Mexican population is the A risk allele of rs8050136, located in the first intron, which has been linked to higher body weight, BMI, and increased dietary energy intake, reinforcing the regulatory significance of this genomic region (Astiazaran-Garcia et al., 2017). Studies suggest that *FTO* variants influence obesity by modulating regulatory activity in adipose tissue; in fact, *FTO* expression has been reported to be elevated in the subcutaneous fat tissue of obese individuals, suggesting a biological mechanism for increased fat storage (Huang et al., 2023; Benak et al., 2024). Furthermore, a study from Kalantari et al. (2018a) proposed that these SNVs act in proximity as a haplotype, potentially affecting nearby gene expression and metabolic pathways (Kalantari et al., 2018b; 2018a). Taken together, the cumulative evidence underscores the central role of *FTO*, particularly rs9939609 and rs8050136, in shaping genetic susceptibility to obesity and the complex interactions among genotype, eating behavior, and energy metabolism.

### 2.2 Dopamine D2 receptor (*DRD2*)

The *DRD2* gene encodes the dopamine D2 receptor, which is part of the dopamine signaling pathway in the brain. Dopamine is a neurotransmitter that plays a key role in reward, motivation, and appetite regulation. Variants in *DRD2* influence reward processing and eating behaviors, contributing to obesity and insulin resistance (Epstein et al., 2007). One of the most relevant genetic variants is the *DRD2/ANKK1* Taq1A variant (rs1800497), in which the A1 allele has been associated with reduced D2 receptor density and increased L-DOPA–related dopaminergic activity (Pohjalainen et al., 1998). This variant has also been linked to altered reward processing and preference for high-calorie foods, which may contribute to overeating and obesity. In a study of Mexican individuals, carriers of the A1 allele showed a higher tendency to consume unhealthy foods and exhibited altered biochemical parameters, including abnormal glucose, triglyceride, and very-low-density lipoprotein (VLDL) levels (Rivera-Iñiguez et al., 2019).

### 2.3 Melanocortin-4 receptor (*MC4R*)

The *MC4R* gene encodes the melanocortin 4 receptor protein, which is part of the melanocortin system in the hypothalamus. This system plays an important role in homeostasis, food intake behavior control (appetite), and energy expenditure by regulating the activation or inhibition of the leptin pathway (HAINER et al., 2020). The activation of *MC4R* suppresses appetite and increases energy expenditure, whereas *MC4R* inhibition increases appetite and reduces energy expenditure (Ayers et al., 2018; Baldini and Phelan, 2019). Loss of function (LoF) variants in the *MC4R* pathway have been shown to cause early-onset severe obesity. These variants alter melanocortin signaling within the leptin–melanocortin pathway, leading to hyperphagia (excessive eating), insulin resistance, reduced energy expenditure, increased BMI, and a higher risk of obesity (Baldini and Phelan, 2019). *MC4R* is now considered a very commonly associated gene with childhood obesity, and its dysfunction has also been linked with diseases such as type 2 diabetes. The rs17782313 analyzed in this study is a non-coding intergenic variant located approximately 190 kb downstream of *MC4R* (Zhang et al., 2023). The C allele variant has a relatively high prevalence in obesity studies in child and adult populations in Mexico (Pérez Herrera, 2018; López-Rodríguez et al., 2020).

### 2.4 Fatty acid-binding protein 2 (*FABP2*)

The *FABP2* gene encodes fatty acid-binding protein 2, which is involved in the absorption and transport of fatty acids in the small intestine; moreover, it plays a role in lipid accumulation and energy homeostasis (Weiss et al., 2002). The Ala54Thr (p.Thr55Ala) variant of the *FABP2* rs1799883 has been extensively studied and is associated with increased intestinal fatty-acid absorption and elevated circulating triglyceride and LDL-cholesterol levels. Consequently, it has been linked to insulin resistance, dyslipidemia, and an increased risk of obesity and type 2 diabetes (Martínez-López et al., 2007; Gonzalez-Becerra et al., 2018).

### 2.5 β2 receptor gene (*ADRB2*)

Catecholamines (adrenaline and noradrenaline) are involved in the regulation of lipolysis in adipocytes via the β-adrenergic signaling pathway. The β2 receptor protein is a seven-transmembrane G protein-coupled receptor encoded by the *ADRB2* gene located at 5q31-32 (Wang and Jiang, 2021). Variants in *ADRB2* modulate β2-adrenergic signaling efficiency and have been associated with obesity and variability in body fat loss in response to physical activity (Kurylowicz et al., 2015; Akinci et al., 2022; de Souza e Silva et al., 2022). In Chilean populations, the genotype AA for the rs12654778 SNV has been associated with lower low-density lipoprotein cholesterol levels (González-Contreras et al., 2024). Some *ADRB2* SNVs associated with obesity and exercise endurance (rs1042713, rs1042714) have been studied in Mestizo populations in Mexico (López-Hernández et al., 2023).

### 2.6 *SH2B1* adaptor protein

The *SH2B1* gene, located at 16p11, encodes an adaptor protein that plays a key role in signaling pathways related to leptin, insulin, and growth factors. Leptin signaling is important because it regulates appetite and energy balance. Additionally, it promotes insulin sensitivity, glucose metabolism, growth and development (Rui, 2014). Variants in *SH2B1*, such as the rs7498665G, have been associated with obesity and metabolic traits. This variant is associated with increased BMI, a higher percentage of body fat, and insulin resistance. On the other hand, loss of function variants in *SH2B1* have been associated with severe obesity, hyperphagia, and metabolic dysfunction in Mexican cohorts (Robayo Zurita, 2023). The A allele of the rs4788102 SNV has been previously related to indices of adiposity and obesity risk and to reduced insulin-stimulated NOS activity (Prudente et al., 2011; Xi et al., 2013).

It is important to mention that although *FTO*, *MC4R*, and *SH2B1* variants have been previously studied in Mexican populations, research on *DRD2*, *ADRB2*, and *FABP2* remains scarce. Given Mexico’s unique genetic admixture, a comprehensive evaluation of these polymorphic variants is needed to better understand population-specific genetic predispositions to obesity. Therefore, this study aimed to determine the genotype and allele frequency distributions of these six obesity- and metabolism-related SNVs in a cohort from Mexico City. We hypothesized that their frequencies would differ from those reported in non-Mexican reference populations and would not vary significantly by sex.

## 3 Materials and methods

We performed a descriptive cross-sectional population genetic analysis to examine the genotype and allele frequency distributions of six obesity- and metabolism-related single nucleotide variants (Table 1) in a cohort of Mexican adults residing in Mexico City. The study population consisted of 129 unrelated participants (59 women and 70 men) with ages ranging from 18 to 87 years. Participants self-identified as Mexican, reporting at least two generations of Mexican ancestry and residence in Mexico City. While ancestry-informative markers were not analyzed, ancestry was characterized based on self-reported data. Given the demographic profile of Mexico City, this cohort is reflective of an urban Mexican-Mestizo population—an admixed group with varying degrees of Indigenous and European genetic contributions.

**TABLE 1:** Selected obesity- and metabolism-related SNVs.

Gene	rsID	Nucleotide change	HGVS c.Nomenclature	HGVS p. Nomenclature
*FTO*	rs9939609	T>A	-	-
*ANKK1/DRD2*	rs1800497	C>T	c.2137G>A	p.Glu713Lys
*MC4R*	rs17782313	T>C	-	-
*FABP2*	rs1799883	A>G	c.163A>G	p.Thr55Ala (Ala54Thr, legacy numbering)
*ADRB2*	rs1042714	G>C	c.79G>C	p.Glu27Gln
*SH2B1*	rs4788102	G>A	-	-

Nucleotide changes are presented using conventional population-based notation, consistent with commonly reported allelic representations for each rsID. HGVS c. and p. nomenclature were reported only for coding variants with a clear and unambiguous impact on the protein sequence; for non-coding or regulatory variants, HGVS notation was omitted to avoid transcript-dependent ambiguity.

Participants were enrolled over a 34-month period using a non-probabilistic convenience sampling strategy within a private clinical genetics setting. The study was conducted in accordance with the ethical principles stipulated in the Declaration of Helsinki, and all participants provided their signed written informed consent.

Given the non-probabilistic convenience sampling strategy, the resulting frequency estimates are intended as descriptive reference data for this Mexico City urban cohort and should not be interpreted as nationally representative.

### 3.1 Inclusion and exclusion criteria

Inclusion: Participants were required to identify as Mexican, report at least two generations of Mexican ancestry, and be residents of Mexico City.

Exclusion: Individuals who were naturalized Mexicans or who reported non-Mexican ancestry within the last two generations.

### 3.2 SNV genotyping and PCR conditions

Genomic DNA (gDNA) was extracted from saliva samples collected using oral swabs using the FlexiGene DNA Kit (Qiagen, Cat. No. 51206) following the manufacturer’s protocol. DNA concentration and purity were assessed by spectrophotometry (A260/A280 = 1.8–2.0) to ensure suitability for allelic discrimination analysis.

SNV genotyping was performed by real-time PCR (qPCR) using TaqMan® SNP Genotyping Assays (Applied Biosystems), which exploit the 5′-nuclease activity of Taq DNA polymerase to detect allele-specific amplification. The specific assays employed in this study were rs9939609 (*FTO*; Assay ID C__30090620_10), rs1800497 (*DRD2*; Assay ID C___7486676_10), rs17782313 (*MC4R*; Assay ID C__32667060_10), rs1799883 (*FABP2*; Assay ID C____761961_10), rs1042714 (*ADRB2*; Assay ID C___2084765_20), and rs4788102 (*SH2B1*; Assay ID C__26672045_10). Each assay includes forward and reverse primers for target amplification, together with two allele-specific minor groove binder (MGB) probes labeled with VIC™ and FAM™, enabling allele discrimination. Genotype assignment was performed using TaqMan Genotyper Analysis Software (v1.4.0), following the manufacturer’s specifications. According to the manufacturer, the TaqMan® SNP Genotyping Assays demonstrate analytical sensitivity and specificity exceeding 99%.

### 3.3 Statistical analysis

Genotypic and allelic frequencies were calculated for each of the six SNVs. Allelic proportions were reported with 95% confidence intervals (CIs) calculated using the Wilson method. Hardy–Weinberg equilibrium (HWE) for each SNV was evaluated using the exact test implemented in the *HardyWeinberg* package of R; chi-square values are reported for descriptive purposes only. Sex-stratified comparisons of allele frequencies were performed using Fisher’s exact test on 2 × 2 contingency tables. When multiple comparisons were conducted, *p*-values were adjusted for false discovery rate (FDR) using the Benjamini–Hochberg procedure. All analyses were performed in R statistical software (v4.x), employing packages including *HardyWeinberg*, *pegas*, and *genetics*.

The study was designed as a descriptive population genetic analysis focused on frequency estimation; no hypothesis-driven association testing or inferential subgroup analyses were conducted.

## 4 Results

### 4.1 Genotypic and allelic frequencies

Six obesity-associated genetic variants were genotyped in the study population: *FTO* rs9939609, *ANKK1/DRD2* rs1800497, *MC4R* rs17782313, *FABP2* rs1799883, *ADRB2* rs1042714, and *SH2B1* rs4788102. The genotypic and allelic frequency distributions for these variants are shown in Tables 2, 3, respectively. Across all SNVs, homozygous genotypes for the most common allele predominated, with the exception of *SH2B1*, which showed a higher proportion of heterozygous genotypes.

**TABLE 2:** Genotypic frequencies of six SNVs associated with obesity.

Genotypic frequencies							
​	​	*n*	freq	*n*	freq	*n*	freq
*FTO*	T>A	T/T	0.48	T/A	0.43	A/A	0.09
rs9939609		**62**		**55**		**12**	
*ANKK1/DRD2*	C>T	C/C	0.47	C/T	0.42	T/T	0.11
rs1800497		**61**		**54**		**14**	
*MC4R*	T>C	T/T	0.77	T/C	0.22	C/C	0.01
rs17782313		**99**		**28**		**2**	
*FABP2*	A>G	A/A	0.56	A/G	0.40	G/G	0.04
rs1799883		**72**		**52**		**5**	
*ADRB2*	G>C	G/G	0.55	G/C	0.40	C/C	0.05
rs1042714		**71**		**51**		**7**	
*SH2B1*	G>A	G/G	0.33	G/A	0.51	A/A	0.16
rs4788102		**43**		**65**		**21**	

Bold values indicate counts (*n*).

**TABLE 3:** Allelic frequencies of six SNVs associated with obesity.

Allelic frequencies							
​	​	*n*	freq	*n*	freq	*x2*	*p*
*FTO*	T>A	T	0.69	A	0.31	0.00	0.96
rs9939609		**179**		**79**			
*ANKK1/DRD2*	C>T	C	0.68	T	0.32	0.15	0.69
rs1800497		**176**		**82**			
*MC4R*	T>C	T	0.88	C	0.12	0.00	0.99
rs17782313		**226**		**32**			
*FABP2*	A>G	A	0.76	G	0.24	1.39	0.23
rs1799883		**196**		**62**			
*ADRB2*	G>C	G	0.75	C	0.25	0.30	0.57
rs1042714		**193**		**65**			
*SH2B1*	G>A	G	0.59	A	0.41	0.18	0.66
rs4788102		**151**		**107**			

Bold values indicate counts (*n*).

Considerable variation in minor allele frequencies (MAF) was observed. The A allele of *SH2B1* (rs4788102) showed the highest MAF (0.41, 95% CI 0.35–0.47), followed by *ANKK1/DRD2* (rs1800497, 0.32, 95% CI 0.27–0.37) and *FTO* (rs9939609, 0.31, 95% CI 0.25–0.36). In contrast, the C allele of *MC4R* (rs17782313) had the lowest MAF (0.12, 95% CI 0.09–0.17), suggesting a limited presence of this variant in the studied cohort. *FABP2* rs1799883 and *ADRB2* rs1042714 showed intermediate MAF values (0.24, 95% CI 0.19–0.30; and 0.25, 95% CI 0.20–0.31, respectively), with genotype distributions consistent with Hardy–Weinberg equilibrium.

All variants conformed to Hardy–Weinberg equilibrium by the exact test (*p* > 0.05), and genotyping call rates exceeded 98%.

### 4.2 Sex-stratified analysis

In the total study population (*N* = 129), 59 females and 70 males were included. Genotypic and allelic frequencies stratified by sex are presented in Tables 4, 5, respectively. Statistical comparisons were performed using Fisher’s exact test as described in the Methods section. No statistically significant differences in genotype or allele distributions were detected between females and males for any of the six variants (*p* > 0.05 after FDR adjustment). These findings indicate that sex did not significantly affect the distribution of the analyzed obesity-associated SNVs, thereby supporting the use of combined analyses for subsequent comparisons.

**TABLE 4:** Genotypic frequencies of six SNVs associated with obesity by sex.

Genotypic frequencies by sex													
​	​	Females (*n* = 59)						Males (*n* = 70)					
		*n*	freq	*n*	freq	*n*	freq	*n*	freq	*n*	freq	*n*	freq
*FTO*	T>A	T/T	0.46	T/A	0.46	A/A	0.08	T/T	0.50	T/A	0.40	A/A	0.10
rs9939609		**27**		**27**		**5**		**35**		**28**		**7**	
*ANKK1/DRD2*	C>T	C/C	0.51	C/T	0.37	T/T	0.12	C/C	0.44	C/T	0.46	T/T	0.10
rs1800497		**30**		**22**		**7**		**31**		**32**		**7**	
*MC4R*	T>C	T/T	0.82	T/C	0.15	C/C	0.03	T/T	0.73	T/C	0.27	C/C	0.00
rs17782313		**48**		**9**		**2**		**51**		**19**		**0**	
*FABP2*	A>G	A/A	0.61	A/G	0.36	G/G	0.03	A/A	0.52	A/G	0.44	G/G	0.04
rs1799883		**36**		**21**		**2**		**36**		**31**		**3**	
*ADRB2*	G>C	G/G	0.61	G/C	0.36	C/C	0.03	G/G	0.50	G/C	0.43	C/C	0.07
rs1042714		**36**		**21**		**2**		**35**		**30**		**5**	
*SH2B1*	G>A	G/G	0.37	G/A	0.49	A/A	0.14	G/G	0.30	G/A	0.51	A/A	0.19
rs4788102		**22**		**29**		**8**		**21**		**36**		**13**	

Bold values indicate counts (*n*).

**TABLE 5:** Allelic frequencies of six SNVs associated with obesity by sex.

Allelic frequencies by sex									
​	​	Female (*n* = 59)				Male (*n* = 70)			
		*n*	freq	*n*	freq	*n*	freq	*n*	freq
*FTO*	T>A	T	0.69	A	0.31	T	0.70	A	0.30
rs9939609		**81**		**37**		**98**		**42**	
*ANKK1/DRD2*	C>T	C	0.70	T	0.30	C	0.67	T	0.33
rs1800497		**82**		**36**		**94**		**46**	
*MC4R*	T>C	T	0.89	C	0.11	T	0.87	C	0.13
rs17782313		**105**		**13**		**121**		**19**	
*FABP2*	A>G	A	0.79	G	0.21	A	0.74	G	0.26
rs1799883		**93**		**25**		**103**		**37**	
*ADRB2*	G>C	G	0.79	C	0.21	G	0.71	C	0.29
rs1042714		**93**		**25**		**100**		**40**	
*SH2B1*	G>A	G	0.61	A	0.39	G	0.56	A	0.44
rs4788102		**73**		**45**		**78**		**62**	

Bold values indicate counts (*n*).

### 4.3 Hardy‒Weinberg equilibrium

All analyzed variants conformed to Hardy–Weinberg equilibrium (HWE), indicating that the observed genotype distributions did not deviate from those expected under equilibrium conditions. No evidence of significant deviation was detected for any SNV by the exact test (*p* > 0.05).

## 5 Discussion

Obesity represents a major global public health challenge. According to published data by the Global Obesity Observatory (World Obesity Federation, 2026), Mexico ranks 34th worldwide with an obesity rate of 32.22%, well below the rates published in the National Health and Nutrition Survey (ENSANUT) (Shamah, 2016) where, just to mention one of the relevant data, the prevalence of abdominal obesity was 76.6%, being higher in women than in men (87.7% vs. 65.4%), and in the 40 to 79 age groups compared to the 20 to 29 age group. These data reflect the magnitude of the problem in the region and underscore the critical need for the development of public policies designed to prevent and reduce this problem. Obesity is recognized worldwide as a multifactorial and heterogeneous condition that arises from the interaction of different factors including genetic, environmental, behavioral, and socioeconomic components. In this context, analyzing the distribution of obesity- and metabolism-related genetic variants in local cohorts provides a necessary foundation for genomic epidemiology studies and may inform future precision medicine strategies tailored to specific populations.

In this descriptive population-based study, we characterized the genotypic and allele frequencies of six widely studied variants linked to obesity (*FTO*, *ANKK1/DRD2*, *MC4R*, *FABP2*, *ADRB2*, and *SH2B1*) in a cohort from Mexico City. The observed frequencies reflect both concordance and divergence when compared descriptively with previously published Mexican cohorts and global reference populations, as summarized in Table 6, consistent with the expected heterogeneity in admixed populations.

**TABLE 6:** Comparative allele frequency landscape across global populations.

Study	*n*	Major allele frequency	Minor allele frequency (MAF)
*FTO* rs9939609			
Central Mexico (Our study)	129	0.6900	0.3100
Western Mexico (Martínez-López et al., 2024)	169	0.6900	0.3100
Western Mexico (Rivera-Iñiguez et al., 2024)	71	0.7000	0.3000
South Mexico (González-Herrera et al., 2019)	303	0.8399	0.1601
Brazil (do Nascimento et al., 2019)	172	0.6395	0.3605
India (Prakash et al., 2016)	333	0.6070	0.3930
Tunisia (Ben Halima et al., 2018)	334	0.6290	0.3710
1,000 Genomes Global	5,008	0.6599	0.3401
1,000 Genomes Africa	1,322	0.5061	0.4939
1,000 Genomes East Asian	1,008	0.8313	0.1687
1,000 Genomes Europe	1,006	0.5865	0.4135
1,000 Genomes South Asian	978	0.7120	0.2880
1,000 Genomes American	694	0.7380	0.2620
*ANKK1/DRD2* rs1800497			
Central Mexico (Our study)	129	0.6800	0.3200
Western Mexico (Rivera-Iñiguez et al., 2019)	276	0.5890	0.4110
Western Mexico (Rivera-Iñiguez et al., 2024)	61	0.6200	0.3800
Southern Mexico (Genis-Mendoza et al., 2017)	123	0.5600	0.4400
Brazil (Cordeiro and Vallada, 2014)	834	0.5905	0.4095
China (Peng et al., 2016)	189	0.6300	0.3700
India (Ghosh et al., 2013)	200	0.6800	0.3200
Egypt (Arab and Elhawary, 2015)	100	0.7150	0.2850
1,000 Genomes Global	5,008	0.6743	0.3257
1,000 Genomes Africa	1,322	0.6150	0.3850
1,000 Genomes East Asian	1,008	0.5942	0.4058
1,000 Genomes Europe	1,006	0.8121	0.1897
1,000 Genomes South Asian	978	0.6850	0.3150
1,000 Genomes American	694	0.6890	0.3110
*MC4R* rs17782313			
Central Mexico (Our study)	129	0.8800	0.1200
Western Mexico (Rivera-Iñiguez et al., 2024)	69	0.8800	0.1200
Central Mexico (García-Solís et al., 2016)	580	0.9020	0.0980
Colombia (Garavito et al., 2020)	155	0.8520	0.1480
Saudi Arabia (Batarfi et al., 2019)	94	0.7770	0.2230
China (Sun et al., 2016)	1,200	0.7050	0.2950
South Africa (Lombard et al., 2012)	969	0.7500	0.2500
1,000 Genomes Global	5,008	0.7600	0.2400
1,000 Genomes Africa	1,322	0.7224	0.2776
1,000 Genomes East Asian	1,008	0.8135	0.1865
1,000 Genomes Europe	1,006	0.7604	0.2396
1,000 Genomes South Asian	978	0.6800	0.3200
1,000 Genomes American	694	0.8660	0.1340
*FABP2* rs1799883			
Central Mexico (Our study)	129	0.7600	0.2400
Western Mexico (Gonzalez-Becerra et al., 2018)	523	0.7300	0.2700
Pakistán (Ijaz et al., 2024)	100	0.5200	0.4800
1,000 Genomes Global	5,008	0.7466	0.2534
1,000 Genomes Africa	1,322	0.7829	0.2171
1,000 Genomes East Asian	1,008	0.7540	0.2460
1,000 Genomes Europe	1,006	0.7316	0.2684
1,000 Genomes South Asian	978	0.6890	0.3110
1,000 Genomes American	694	0.7690	0.2310
*ADRB2 rs1042714*			
Central Mexico (Our study)	129	0.7500	0.2500
Central Mexico (López-Hernández et al., 2023)	282	0.8848	0.1152
Brazil (Santos et al., 2022)	70	0.7210	0.2790
China (Guo et al., 2016)	340	0.7000	0.3000
Taiwan (Hsiao and Lin, 2014)	502	0.9073	0.0927
1,000 Genomes Global	5,008	0.7957	0.2043
1,000 Genomes Africa	1,322	0.8638	0.1362
1,000 Genomes East Asian	1,008	0.9266	0.0734
1,000 Genomes Europe	1,006	0.5905	0.4095
1,000 Genomes South Asian	978	0.8070	0.1930
1,000 Genomes American	694	0.7580	0.2420
*SH2B1* rs4788102			
Central Mexico (Our study)	129	0.5900	0.4100
Arabia Saudita (Al-Hakeem, 2014)	300	0.7900	0.2100
1,000 Genomes Global	5,008	0.7394	0.2606
1,000 Genomes Africa	1,322	0.7753	0.2247
1,000 Genomes East Asian	1,008	0.8740	0.1260
1,000 Genomes Europe	1,006	0.6710	0.3290
1,000 Genomes South Asian	978	0.7820	0.2180
1,000 Genomes American	694	0.5140	0.4860

1. Percentages sum may not total 1 due to rounding in the original source.

2. Sample size corresponds to the number of successfully genotyped individuals as reported in genotype tables.

3. For cross-study comparability, allele frequencies are presented as major and minor (MAF), as allele letter conventions and reference strands may differ across publications and genotyping platforms.

4. 1,000 Genomes reference population allele frequencies were retrieved from dbSNP (NCBI, 2026).

Regarding the rs9939609 T>A variant of *FTO*, we observed in our cohort a high proportion of heterozygous individuals (T/A = 43%), and an A allele frequency of 0.31. These values are broadly consistent with data reported in studies conducted in other mestizo Mexican populations, which also highlight the considerable regional heterogeneity for this variant. Notably, the minor allele frequency observed in our cohort (0.31) is identical to that reported in a Western Mexico Mestizo sample (Martínez-López et al., 2024) and highly similar to another Western Mexico cohort (Rivera-Iñiguez et al., 2024), whereas a Southern Mexico sample (González-Herrera et al., 2019) reported a substantially lower frequency (0.16), illustrating regional heterogeneity within Mexican populations. Recent evidence from Mexican populations suggests that the phenotypic impact of rs9939609 may vary depending on genetic background and population structure. In a large cross-sectional study that included indigenous and mestizo groups, the A allele was more frequent in mestizo populations, while indigenous groups showed very high frequencies of the T allele. In the case of mestizo populations, there was no significant association with body mass index, although genotype-based differences in metabolic parameters such as triglycerides, insulin, and glucose levels were detected. This suggests that the effects of this variant may extend beyond obesity *per se* and may not be uniform across different populations. At the continental level, 1,000 Genomes data show lower frequencies in East Asian populations and comparatively higher frequencies in European and African groups, reflecting broad global variability at this locus (NCBI, 2026). These findings support the interpretation that the distribution of rs9939609 in Mexican populations is determined by ancestry and admixture, and that its metabolic relevance may depend on the specific contexts of each population. In line with the descriptive nature of the present study, our results contribute to the data on the frequency of this variant in an urban cohort in Mexico City, without implying direct phenotypic associations or causal effects (Sepulveda-Villegas et al., 2025).

With respect to *ANKK1/DRD2* rs1800497 (Taq1A), we observed genotype frequencies of 0.47 for C/C, 0.42 for C/T, and 0.11 for T/T in our Mexico City cohort. Rivera-Iñiguez et al. reported genotype frequencies of 36.2% for A2A2 (C/C), 45.3% for A1A2 (C/T), and 18.5% for A1A1 (T/T) in a larger sample of Mexican mestizo adults. Although their study focused on dietary behavior and biochemical traits, these genotype distributions provide a useful descriptive reference and are broadly comparable to our observed pattern, supporting regional and sampling-related variability in rs1800497 within Mexican populations. In both cohorts, the homozygous genotype (C/C) was more frequent than the homozygous genotype (T/T), while heterozygotes represented an intermediate proportion (Rivera-Iñiguez et al., 2019). In addition, allele frequency comparisons with 1,000 Genomes populations show that the minor allele frequency in our cohort falls within the variability range observed in global reference populations, further supporting the descriptive consistency of this locus across diverse ancestries (NCBI, 2026).

With respect to *MC4R* (rs17782313), the C allele showed the lowest MAF among the six loci in our cohort (0.12; 95% CI 0.09–0.17). The minor allele frequency observed in our sample is identical to that reported in a Western Mexico cohort (Rivera-Iñiguez et al., 2024), further supporting regional consistency of this locus across geographically distinct Mexican populations. When contrasted with prior Mexican data, León-Mimila et al. reported a markedly lower C-allele frequency for rs17782313 in Mexican-mestizo adults (RAF ≈ 7.3%) and an even lower frequency in indigenous adults (RAF ≈ 1.52%). The higher C-allele MAF observed in our Mexico City cohort may reflect differences in sampling frame, regional genetic structure, and admixture patterns across Mexican subpopulations; therefore, broader probabilistic sampling will be required to determine whether these differences persist at the population level (León-Mimila et al., 2013). Pediatric cohorts have also reported variability of rs17782313 across Mexican children with different ancestry backgrounds, although direct comparisons with adult convenience samples should be interpreted cautiously (López-Rodríguez et al., 2020). In the broader global context, 1,000 Genomes data show intermediate minor allele frequencies for rs17782313 in European populations and comparatively higher frequencies in several African and South Asian groups, whereas East Asian populations tend to exhibit lower values (NCBI, 2026). The MAF observed in our cohort (0.12) falls within this global variability spectrum, supporting its descriptive consistency across admixed populations.

With respect to *FABP2* rs1799883 (Ala54Thr; legacy numbering), we observed genotype frequencies of 0.56 for A/A, 0.40 for A/G, and 0.04 for G/G in our Mexico City cohort, corresponding to a minor allele frequency of 0.24. Comparable population-based data are available from González-Becerra et al., who reported genotype frequencies of 52.2% for Ala54Ala, 41.1% for Ala54Thr, and 6.7% for Thr54Thr, with a Thr54 allele frequency of 27% in a larger Mexican sample (Gonzalez-Becerra et al., 2018). Despite differences in study design and clinical focus, the overall genotype distribution pattern is broadly similar between both cohorts, with the A/A homozygous genotype being the most frequent, followed by heterozygotes, and a low proportion of G/G homozygotes. These findings support the presence of a consistent population-level distribution of *FABP2* rs1799883 in Mexican cohorts and provide reference frequency data for this variant in an urban population from Mexico City, without implying phenotypic associations or causal effects. Similarly, comparison with 1,000 Genomes reference data indicates that the minor allele frequency observed in our cohort (0.24) is comparable to global estimates across multiple continental populations, although higher frequencies have been reported in certain South Asian cohorts (NCBI, 2026), including a Pakistani sample (0.48) (Ijaz et al., 2024) and the 1,000 Genomes South Asian population (0.31) (NCBI, 2026). These differences illustrate intercontinental variability while supporting the interpretation of rs1799883 as a common variant with stable population-level distribution patterns across diverse ancestries.

With respect to *ADRB2* rs1042714 (Gln27Glu), we observed genotype frequencies of 0.55 for G/G, 0.40 for G/C, and 0.05 for C/C in our Mexico City cohort, corresponding to a minor allele frequency of 0.25. Comparable population-based data from Mexican Mestizo individuals have been reported by López-Hernández et al., who observed a markedly higher prevalence of the C/C genotype and a lower proportion of G/G carriers. Despite differences in sampling design and population structure, both datasets highlight substantial variability in the distribution of rs1042714 across Mexican cohorts. In the broader global context, 1,000 Genomes data demonstrate substantial continental variability for this SNV, with markedly lower minor allele frequencies in East Asian populations and higher frequencies in European populations (NCBI, 2026). The frequency observed in our cohort falls within this global variability spectrum, reinforcing the influence of population structure and sampling design in admixed populations. Taken together, these findings underscore the relevance of regional and sampling-related factors when interpreting *ADRB2* genotype frequencies and support the value of reporting population-specific reference data, without implying phenotypic associations or causal effects (López-Hernández et al., 2023).

With respect to *SH2B1* rs4788102, the frequency of the A allele in our cohort was 0.41. Given the limited number of published data for this variant in Mexican cohorts, we contextualized this finding using public population frequency data available through the NCBI dbSNP database (NCBI, 2026), where considerable variability across ancestry groups is reported, including relatively high frequencies of the A allele in American populations. Consistent with this observation, the minor allele frequency in our cohort (0.41) is close to that reported in the 1,000 Genomes American reference population (0.486), while differing from several non-American continental groups (NCBI, 2026). These comparisons are intended as descriptive benchmarks rather than formal hypothesis testing and emphasize the importance of generating region-specific frequency data for variants previously implicated in obesity-related traits.

By analyzing these variants collectively, the selected loci can be organized into interconnected biological pathways that contribute to energy homeostasis and metabolic regulation. Variants in *FTO*, *MC4R*, and *SH2B1* are involved in central appetite regulation and leptin-melanocortin signaling; *FABP2* participates in the absorption of intestinal fatty acids and lipid handling; *ADRB2* influences adrenergic-mediated lipolysis and energy expenditure; and *ANKK1/DRD2* is related to dopaminergic reward processing and the modulation of eating behavior. Although the present study was not designed to evaluate functional or phenotypic effects, the allelic distribution observed through these complementary pathways underscores the multifactorial influence of the genomic architecture underlying obesity-related phenotypes.

Our results highlight the considerable heterogeneity among the Mexican population, which can be explained by its complex demographic history, indigenous ancestry, European admixture, as well as migration patterns, which reinforce the critical need to conduct regional genomic epidemiological studies across the country. Our findings document the frequency distribution of common SNVs previously reported in association with obesity-related traits within this urban Mexico City cohort. While these variants have been studied in metabolic contexts, the present analysis was designed exclusively to characterize population-level genotype and allele frequencies. From a population genetics perspective, genetic variation represents one component within a broader multifactorial framework that includes environmental, behavioral, and sociocultural determinants. Therefore, the frequencies reported here should be interpreted as descriptive reference data rather than as indicators of individual-level risk or phenotypic prediction. Within this framework, documenting the frequency landscape of common SNVs previously reported in metabolic contexts may inform future integrative studies, including polygenic modeling approaches and nutrigenomic research strategies tailored to admixed urban populations.

## 6 Limitations and future perspectives

Given the relatively modest sample size and the non-probabilistic sampling strategy, the results should be interpreted carefully. The use of convenience sampling within a private clinical genetics setting may introduce selection bias and limit representativeness relative to the broader Mexico City population. In addition, ancestry was determined by self-report in the absence of ancestry-informative markers, which may not fully capture the complex admixture structure of the Mexican population. Furthermore, the lack of detailed phenotypic data (e.g., BMI and metabolic parameters) precludes genotype–phenotype evaluation. These factors should be considered with caution when interpreting cross-population comparisons with previously published studies and public genomic databases, as differences in sampling strategies, population composition, and methodologies may influence the observed allele frequencies. Accordingly, the allele frequency estimates reported here are intended as descriptive reference data for this urban Mexico City cohort and should not be extrapolated to the Mexican population as a whole or to other regions of the country without confirmation in probabilistically sampled studies. Further research involving larger and more diverse cohorts across different Mexican regions will be essential to validate these findings and to explore gene–environment interactions that modulate the effects of these variants. Integrating genomic data with detailed clinical and phenotypic information may provide a more comprehensive understanding of how these variants influence individual metabolic risk and could facilitate the development of precision strategies for obesity prevention and metabolic health management.

## 7 Conclusion

The results of the distribution of allelic and genotypic frequencies of the obesity-associated genetic variants analyzed in our study contribute to a better understanding of the genomic landscape that may influence vulnerability to obesity-related phenotypes in the Mexican population. While further validation is required in larger and more diverse cohorts—considering the genomic diversity among population-specific groups—our findings may serve as a basis for future studies focused on gene-environment interactions, which could contribute to the design and incorporation of precision genomic medicine strategies in public and private health systems, and potentially support the development of public health policies.

## Data Availability

The original contributions presented in the study are included in the article/supplementary material, further inquiries can be directed to the corresponding author.
